# Collecting water: Shashemene, Ethiopia, November 2015

**DOI:** 10.3402/gha.v9.31958

**Published:** 2016-05-04

**Authors:** Hilary Bambrick, Stefano Moncada

**Affiliations:** 1 School of Medicine, Western Sydney University, h.bambrick@westernsydney.edu.au; 2 Institute for European Studies, University of Malta, stefano.moncada@um.edu.mt

**Figure:**
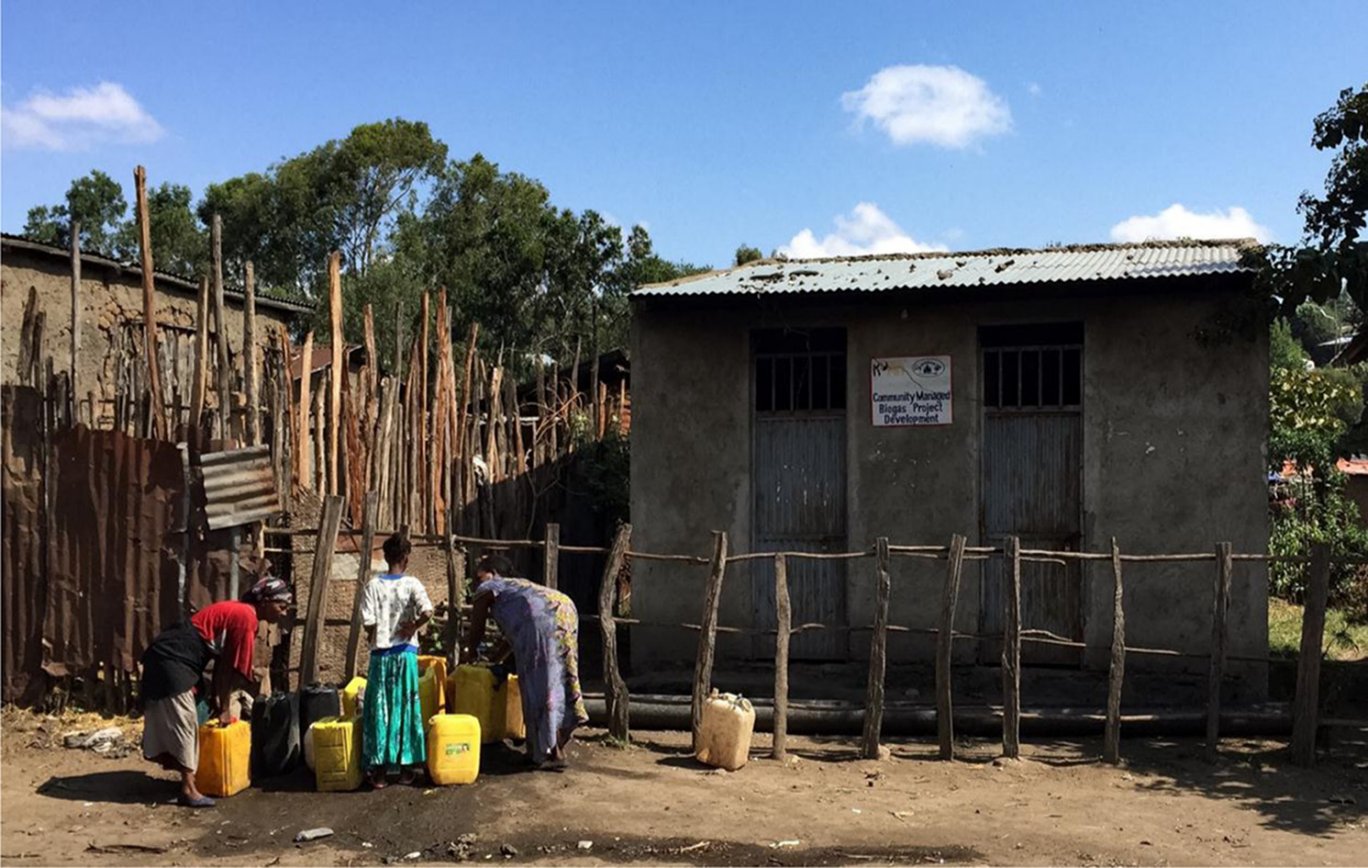


A water point was installed in 2012 as part of a biogas development initiative in an informal urban community of 200 households. The facility provides sanitation, gas for cooking, and organic fertiliser. It consists of four latrines and an inlet for animal waste, a fermentation chamber where the biogas (methane) is produced, and a communal kitchen with four stoves. The slurry by-product is rendered safe by fermentation and used as fertiliser for the community garden.

This facility solves the problem of managing human and animal waste in the community, thereby reducing gastroenteric disease. The methane produced provides a much cleaner alternative cooking fuel to solid biomass fuels such as wood, dung, and charcoal. This leads to reduced exposure to indoor air pollution, lowering the risk of associated respiratory disease. Yet another benefit is that families are able to reduce the amount of money spent on fuel. The fertiliser produced by the system facilitates community food production, increasing food security and generating income.

The water is piped from the town and is sold more cheaply than water from external vendors, and is much cleaner than the water from the small river that trickles through the community used for washing and toileting. The money raised by the community through the sale of water to its members is used to maintain the biogas facility, ensuring its sustainability.

This community typifies many in the region with its poor health, poverty, and vulnerability to climate change. Biogas is a climate-compatible development that has the potential not only to directly improve health through better sanitation and reduced exposure to dangerous indoor air pollution but also to alleviate poverty through associated income generation and reduced expenditure on food and fuel.

The 2015–2016 El Niño drought in Ethiopia highlights the urgency of building resilience through projects like this in communities most at risk due to climate change.

*Hilary Bambrick* School of Medicine Western Sydney University h.bambrick@westernsydney.edu.au *Stefano Moncada* Institute for European Studies University of Malta stefano.moncada@um.edu.mt
